# Analytical development and optimization of a graphene–solution interface capacitance model

**DOI:** 10.3762/bjnano.5.71

**Published:** 2014-05-09

**Authors:** Hediyeh Karimi, Rasoul Rahmani, Reza Mashayekhi, Leyla Ranjbari, Amir H Shirdel, Niloofar Haghighian, Parisa Movahedi, Moein Hadiyan, Razali Ismail

**Affiliations:** 1 Centre for Artificial Intelligence and Robotics, Universiti Teknologi Malaysia, 54100 Kuala Lumpur, Malaysia; 2 Malaysia Japan International Ins. Of Technology, Universiti Teknologi Malaysia, 54100 Kuala Lumpur, Malaysia; 3 Faculty of Electrical Engineering, khayyam higher education Institute, 9189747178, Mashhad, Iran; 4 Department of Mathematical Science, Faculty of Science, Universiti Teknologi Malaysia, 81310 UTM, Johor Bahru, Malaysia; 5 Department of Chemical Engineering, Åbo Akademi University, 20500 Åbo, Finland; 6 Department of physics and CNISM, University of Genova, Via Dodecaneso 33, 16146 Genova, Italy; 7 Department of Information Technology, University of Turku , 20014 Turku, Finland; 8 Department of Electrical and computer engineering, K. N. Toosi University of Technology, Tehran, Iran; 9 Faculty of Electrical Engineering, Universiti Teknologi Malaysia, 81310, UTM Johor Bahru, Johor, Malaysia

**Keywords:** analytical modeling, ant colony optimization (ACO), electrolyte-gated transistors (EGFET), graphene, quantum capacitance

## Abstract

Graphene, which as a new carbon material shows great potential for a range of applications because of its exceptional electronic and mechanical properties, becomes a matter of attention in these years. The use of graphene in nanoscale devices plays an important role in achieving more accurate and faster devices. Although there are lots of experimental studies in this area, there is a lack of analytical models. Quantum capacitance as one of the important properties of field effect transistors (FETs) is in our focus. The quantum capacitance of electrolyte-gated transistors (EGFETs) along with a relevant equivalent circuit is suggested in terms of Fermi velocity, carrier density, and fundamental physical quantities. The analytical model is compared with the experimental data and the mean absolute percentage error (MAPE) is calculated to be 11.82. In order to decrease the error, a new function of *E* composed of α and β parameters is suggested. In another attempt, the ant colony optimization (ACO) algorithm is implemented for optimization and development of an analytical model to obtain a more accurate capacitance model. To further confirm this viewpoint, based on the given results, the accuracy of the optimized model is more than 97% which is in an acceptable range of accuracy.

## Introduction

The astonishing discovery of graphene as an extraordinary two-dimensional (2D) material with low dimensional physics, and possible applications in electronics [1–6] has attracted the attention of scientists in these days. Geim, in 2004, demonstrated that the six-membered rings are the basis of all carbon materials in electrochemical biosensor research [7]. The remarkable electrical properties of graphene such as fast electron transport, tunable energy bandgap, high thermal conductivity, and ballistic transport at room temperature give rise to the potential applicability in electrolyte-gated transistors [8–11]. Graphene, as a nearly perfect 2D crystal free of the structural defects [12–13] shows ballistic transport because of its significant high electron mobility at low temperatures, which can reach up to 200,000 cm^2^/V·s with a typical carrier concentration of 2·10^11^ cm^−2^ [7,14]. Recently attempts have also been made to use graphene as a novel channel material in field effect transistors (FETs) for electronics [15]. The remarkable properties of graphene reported so far included high stiffness with a Young’s modulus of approximately 1000 GPa, a significant heat conductivity of 3000 W·(m·K)^−1^, and large specific surface area of 2600 m^2^·g^−1^ [15–17]. Intrinsic graphene is a semi metal or a zero band gap semiconductor, which results in a high electron mobility at room temperature [18]. Therefore, the electron transfer in graphene is expected to be 100 times faster than that in silicon. Other advantages of graphene, which make it a perfect semiconductor is its massless Dirac fermion structure with zero band gap (graphene is considered to be theoretically lossless) [19]. Compared to silicon-based devices, graphene with its outstanding properties such as consuming less energy and faster heat dissipating show a great promise in electrolyte-gated graphene ﬁeld-effect transistors (EGFETs) [20].

**Figure 1:**
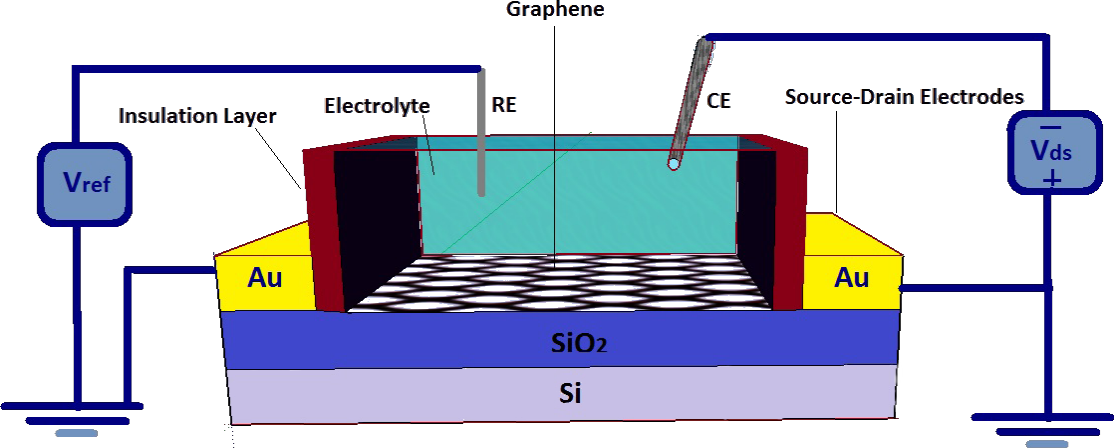
A schematic of a graphene-based EGFET including the bias configuration (three-electrode electrochemical cell).

An EGFET fabricated on a SiO_2_/Si substrate with gold source and drain electrodes and a graphene layer as a conducting channel can be seen in Figure 1. A 300 nm SiO_2_ layer as a back-gate dielectric has been deposited above the doped silicon substrate. The graphene layer, the gate, and a quasi-reference electrode were covered by a small droplet of ionic liquid [21]. The standard three-electrode electrochemical cell using a potentiostat has measured the interfacial capacitance of the graphene [22]. *V*_G_ is the voltage applied at the Pt gate electrode, and *V*_ref_ is the voltage measured on the quasi-reference electrode. Ye et al. have discussed the distinguished properties and high carrier-density transport of ion-gated mono-, bi-, and trilayer graphene bases in double-layer transistors [23]. They demonstrated that *V*_ref_ ≈ 0 over the whole sweep range of *V*_G_, which has led to dropping all the applied *V*_G_ at the liquid/graphene interface [15].

**Figure 2:**
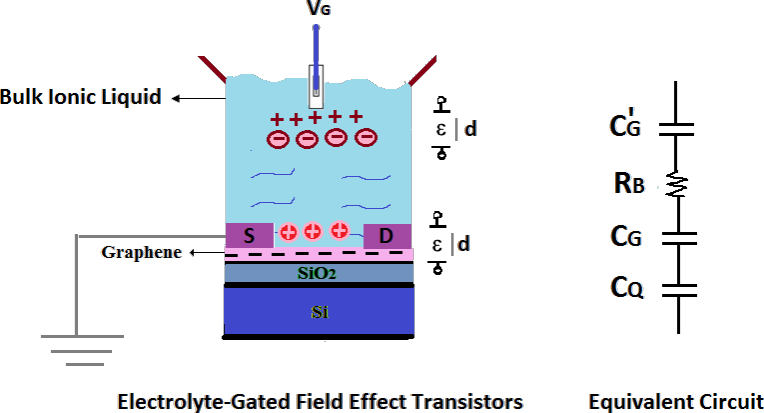
A cross-section of graphene-based electrolyte-gated field effect transistor, together with the equivalent electrical circuit.

In order to minimize the background capacitance,the mentioned configuration is employed which can also prevent the graphene edges from exposure to the electrolyte. To interpret the electrical response of the device, an analytical model of the EGFET together with the equivalent circuit describing its operation is discussed in this paper. As depicted in Figure 2, the measured capacitance is assumed as the contribution of two interfacial capacitances which arise from the double layer formed by ions at the graphene–ionic liquid interface and the quantum capacitance of graphene. The particular case of EGFET is discussed in the context of 2D systems.

To substantiate all the consideration just made, the equivalent circuit of the EGFET device is suggested in terms of a simple, well-defined theoretical model. It is seen that *C*′_G_ is the capacitance that forms between the gate and the ionic liquid, *R*_B_ is the electrical resistance of the solution, *C*_G_ represents the geometrical capacitance of the double layer/graphene interface to model the accumulation of a layer of counter-ions on a charged electrode. Finally, *C*_Q_ is the quantum capacitance of the EGFET associated with the finite density of states of graphene [24]. Figure 2 shows that *V*_G_ has a strong influence on the capacitance. The total capacitance is given by 1/*C* = 1/*C*_G_ + 1/*C*_Q_ with the smaller of the two capacitances dominating the total capacitance. Previous experimental studies have reported a large geometrical capacitance (several tens of μF∕cm^2^) [25–26]. Since the two mentioned capacitances are connected in series, the smallest one would dominate the total capacitance. Hence, geometrical capacitance is neglected compared to the theoretically predicted quantum capacitance of graphene. As expected, *C*_Q_ dominates the total capacitance, which is why the position of the Fermi energy *E*_F_ can be tuned by applying only small values of *V*_G_. These explanations of the current study are consistent with those of Jilin Xia [26] in 2009, who found that the Debye ionic screening length of the ionic liquid is virtually zero, which makes the quantum capacitance a dominant source of the measured capacitance. They performed the measurement of quantum capacitance of bilayer graphene in an ionic liquid electrolyte. The aim of this study is to evaluate the quantum capacitance of single layer graphene sheet as a function of voltage, and validate theoretical predictions with the experimental results [26].

## Results and Discussion

### Proposed model

The quantum capacitance of nanoscale devices is considered as an important quantity in the design of nanoelectronic devices. The classic expression for quantum capacitance utilized in the prediction of the theoretical model for an ideal single layer graphene [27–28] is

[1]
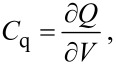


in which ∂*Q* = e·∂*n* is the charge measured in coulombs, *e* is the electron charge, and *n* is the intrinsic carrier concentration of graphene. By substitution of the applied voltage ∂*V* = ∂*E*/*e* to the device we obtain

[2]
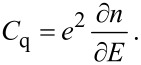


In the modeling process, the density of state (DOS) and the Fermi probability function, *f*(*E*), are employed. It is notable that electrical property of materials from metal to semiconductor is changing by the gradient of DOS(*E*) near the Dirac point [29].

[3]
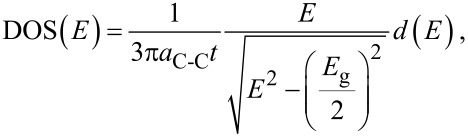


where *a*_C-C_ = 1.42 Å is the carbon–carbon bond length, *t* = 2.7 eV is the nearest neighbor C–C tight binding overlap energy,


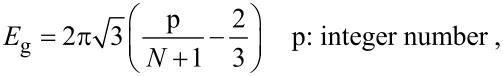


is the energy band gap of graphene monolayer, and the Fermi probability function *f*(*E*) is defined as [30]

[4]
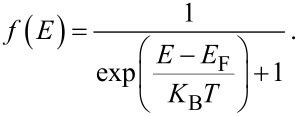


The integral of these two values with respect to *E* gives the carrier concentration equation as

[5]
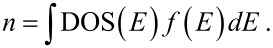


By replacing the DOS and *f*(*E*) in Equation 3, the carrier concentration in the non-parabolic region is defined as

[6]
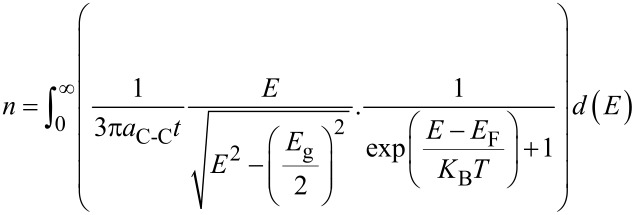


Now the quantum capacitance can be calculated as:

[7]
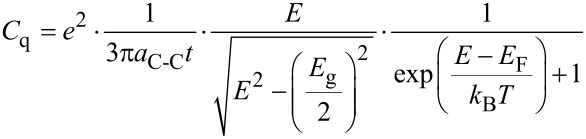


**Figure 3:**
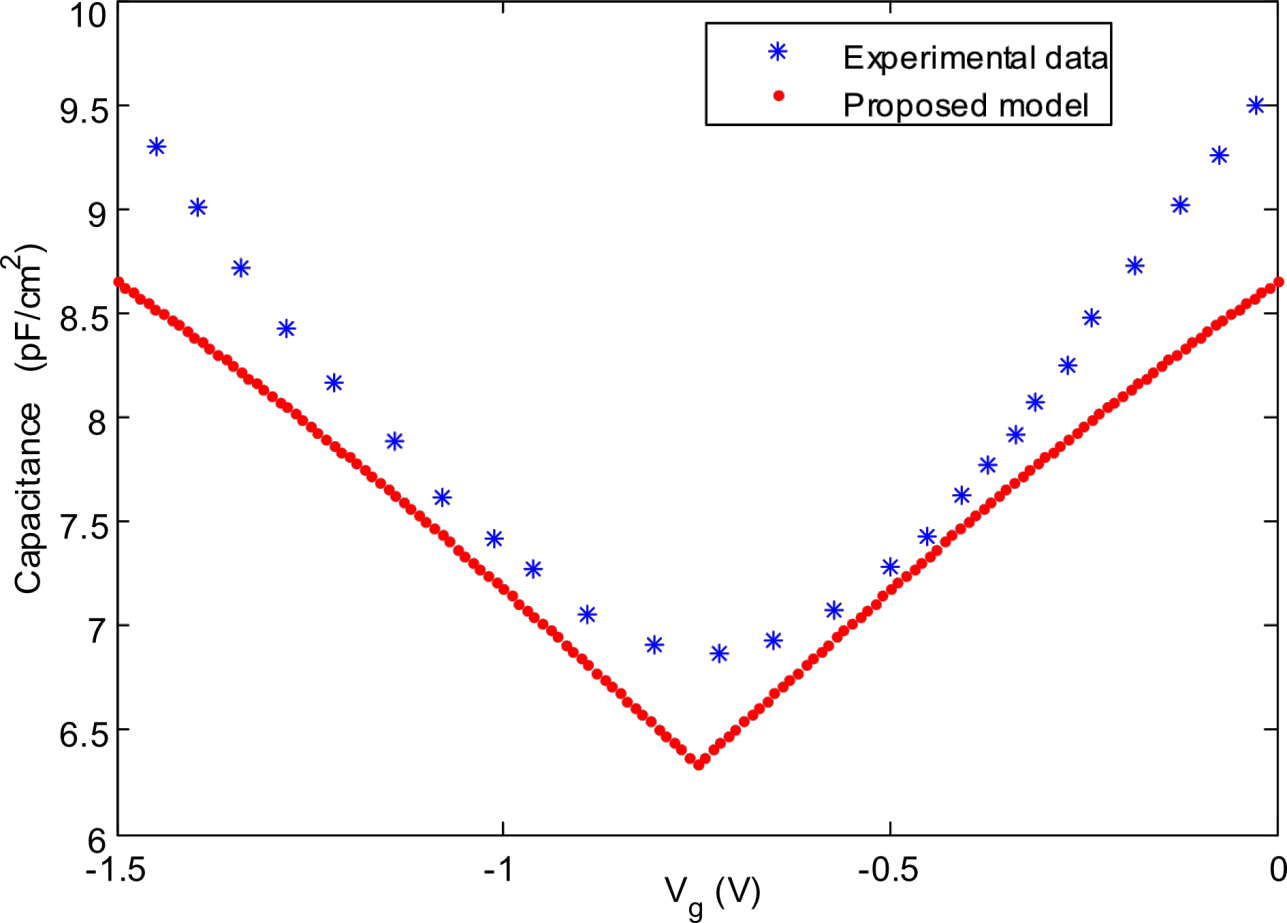
The proposed model of quantum capacitance of EGFETs based single-layer graphene.

The equation provides a quantitative description of the graphene quantum capacitance in terms of the Fermi velocity [31], carrier density, temperature and fundamental physical quantities. According to the relationship between energy band structure and the graphene potential, the quantum capacitance–voltage characteristic of the proposed model is depicted in Figure 3. In the figure, the experimental data is also plotted to have a fair scale for validating the proposed model [32].

To get a greater insight into the quantum capacitance of graphene-based EGFET devices, a number of important characteristics of the *C*–*V* curve are discussed. To begin with, at the Dirac point, the quantum capacitance has a minimum value which is close to zero. The other evident aspect in the quantum capacitance model is the linear rise of the capacitance with the voltage, which is symmetric with respect to the Dirac point. Despite of all mentioned vintages of the proposed model, the characteristics of the proposed model diverge considerably from the experimental data. To ease this error, a new function of *E* is multiplied by the previous model in Equation 7. This function is a square root function, which must be symmetric to its own origin. Otherwise, it will disrupt the isochronism of the proposed model. Equation 8 shows the general form of the suggested function multiplied by the presented model in Equation 7. The quantum capacitance, *C*_q_, is now

[8]
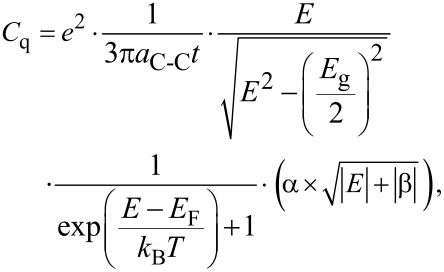


where α and β are unknown parameters, which need to be adjusted properly. Finding the best fitted values for α and β requires an optimization technique with an accurate and reliable performance. To this end, ant colony optimization (ACO) is used as one of the well-known and efficient metaheuristic swarm intelligence-based optimization algorithms. The ACO algorithm has several advantages over conventional mathematic algorithms [33–34]. It is a fast converging algorithm with the capability of escaping from local optima in the search space. The random values used in the movements of the particles help the algorithm to stochastically improve the obtained solution during each iteration.

### Ant colony optimization overview

Ant colony optimization (ACO), which is inspired by the foraging behavior of real ant colonies exploring for foods, was proposed by Dorigo in 1992 [33,35–36]. ACO mainly imitates the team work of an ant colony in finding a food source. If an ant finds a food source, it will carry a portion of the food to the nest, after performing some evaluations about the size of the source. On the way back to the nest, it releases some pheromones, which are known as pheromone trail. The rest of the ants in the nest can reach to the food source by tracing the remaining pheromones. The same behavior is shown by the rest of the ants in returning to the nest from the source. The amount of the pheromones deposited on the way is quite dependant on the quantity and quality of the food source [37]. The pheromone (τ_t_) is a vaporizable substance and its amount decreases over time. Therefore, the path with the highest amount of pheromone is the one, which was chosen by the more ants than the other paths. The shortness of the path is a priority to the pheromone trail and the ants try to find the shortest possible path. In the ACO technique, the pheromone trail that represents a better solution, is updated consequently and there exist several ant colony models in the literature [37–39]. Equation 9 presents the location of the *k*-th ant in the solution space:

[9]
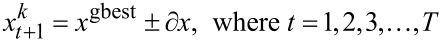


where *T* is the total number of iterations and t denotes the iteration number, *x*^gbest^ is the location of the best objective value obtained until iteration *t*, and ∂*x* is a random vector generated from [−α, α] to determine the allowed variation the ant can have from the *x*^gbest^ with the same dimensions. The length of this jump (variation) is obtained from Equation 10 at the end of √*T* iterations.

[10]
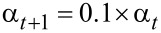


The direction of the variation from *x*^gbest^, which is shown by ± in Equation 9 is decided based on the following equation:

[11]
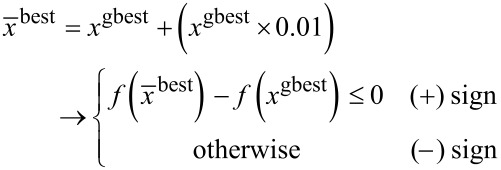


To simulate the evaporation of the pheromone, Equation 12 is presented, and Equation 13 shows the increment of the pheromone around the best objective value obtained after each iteration.

[12]
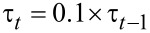


[13]
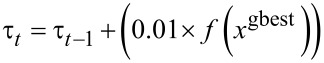


### Optimization of the proposed model

**Figure 4:**
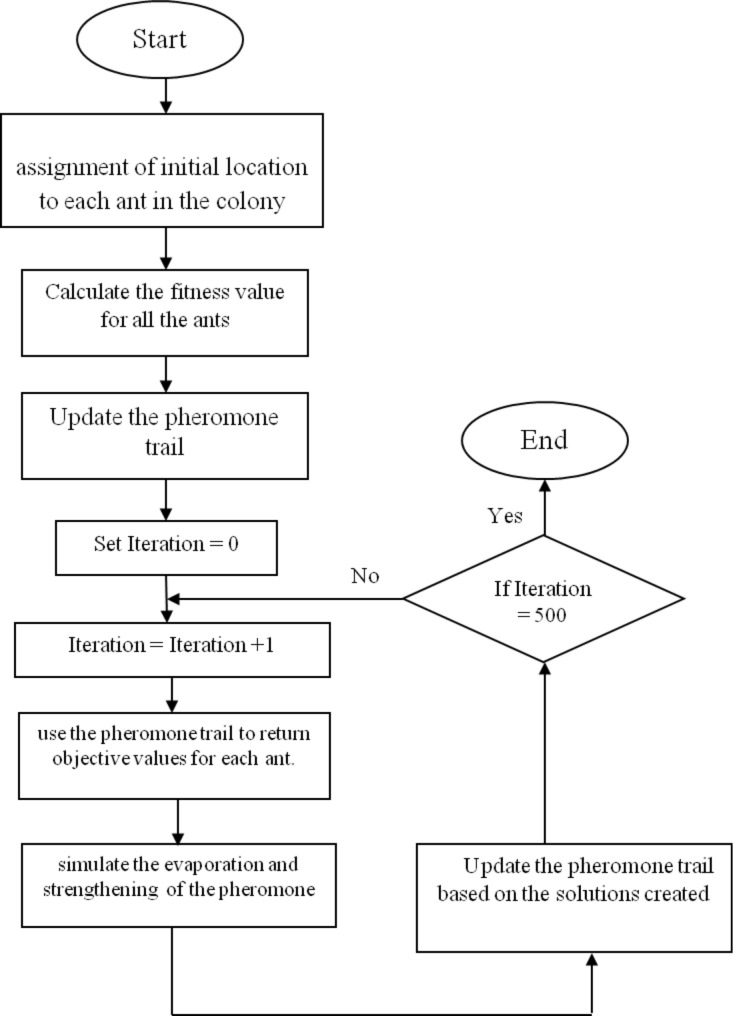
A flowchart of ACO-based algorithm for optimizing the quantum capacitance model.

**Figure 5:**
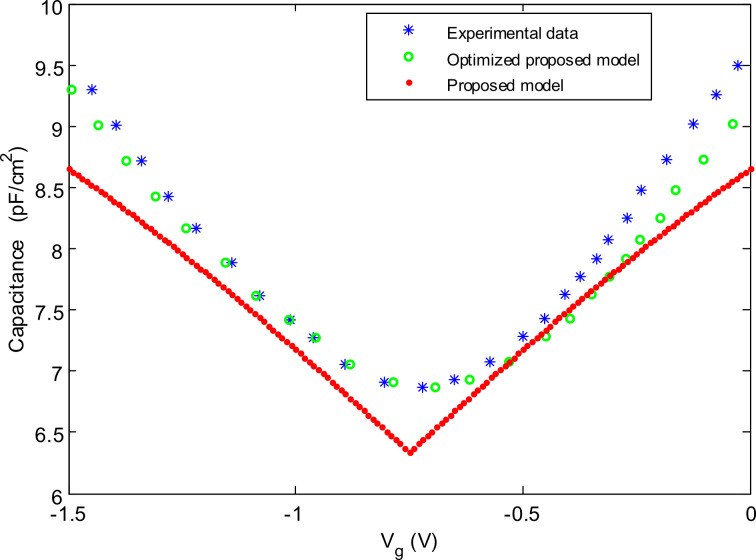
Comparison between the proposed single-layer graphene quantum capacitance model, the optimized proposed model and the experimental extracted data.

The aim of the optimization is to find the best values for α and β in Equation 8. Therefore, the search space of the problem is a 2D space, which returns values for α and β at each iteration. The number of ants hired as agents in the ACO algorithm is set to be 100, which requires a matrix of 100 × 2 to store the α and β values for all the ants, at each iteration. To evaluate the solutions proposed at each iteration, a fitness function is defined as:

[14]
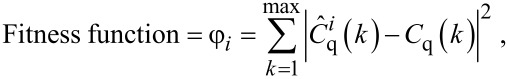


where 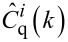 represents the modelled quantum capacitance waveform for particle *i*, *C*_q_(*k*) is the experimental data of the quantum capacitance, and φ*_i_* is the fitness value for the *i*-th ant in the colony*.* The chosen fitness function calculates the squared error between the proposed model and experimental data; hence, the lowest value for the fitness function indicates the best solution to the α and β values. The strategy of the ACO algorithm for the optimization is shown in Figure 4 as a flow chart. The best values obtained for the parameters α and β after the optimization process are shown in Table 1. The fitness value that is the best of 30 runs of the algorithm, and the respective values for the desired parameters are tabulated. Figure 5 shows the proposed single-layer graphene quantum capacitance model, the optimized proposed model and the experimental extracted data, as graphs.

**Table 1:** The best values of the optimized parameters over the 30 runs.

number of runs	maximum iteration number	best fitness value	optimized value for α	optimized value for β

30	10,000	3.289·10^−6^	1.0753	0.724

To evaluate the quality of the optimized model compared with the experimental data, the mean absolute percentage error (MAPE) is used as an error evaluation parameter, as shown in Equation 15.

[15]
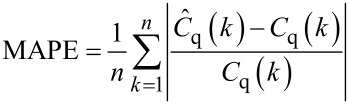


The results for the MAPE for the proposed model and the optimized proposed model are shown in Table 2. The accuracy based on the obtained MAPE value is also reported as the result of subtracting the MAPE from 100 percent. Based on the results tabulated, the accuracy of the optimized model is more than 97%, which is in an acceptable range of accuracy.

**Table 2:** The MAPE value of the optimized proposed single layer graphene quantum capacitance model.

capacitance vs voltage characteristic	MAPE value (%)	accuracy based on MAPE (%)

optimized proposed model	2.54	**97.46**
proposed model	11.82	88.18

**Figure 6:**
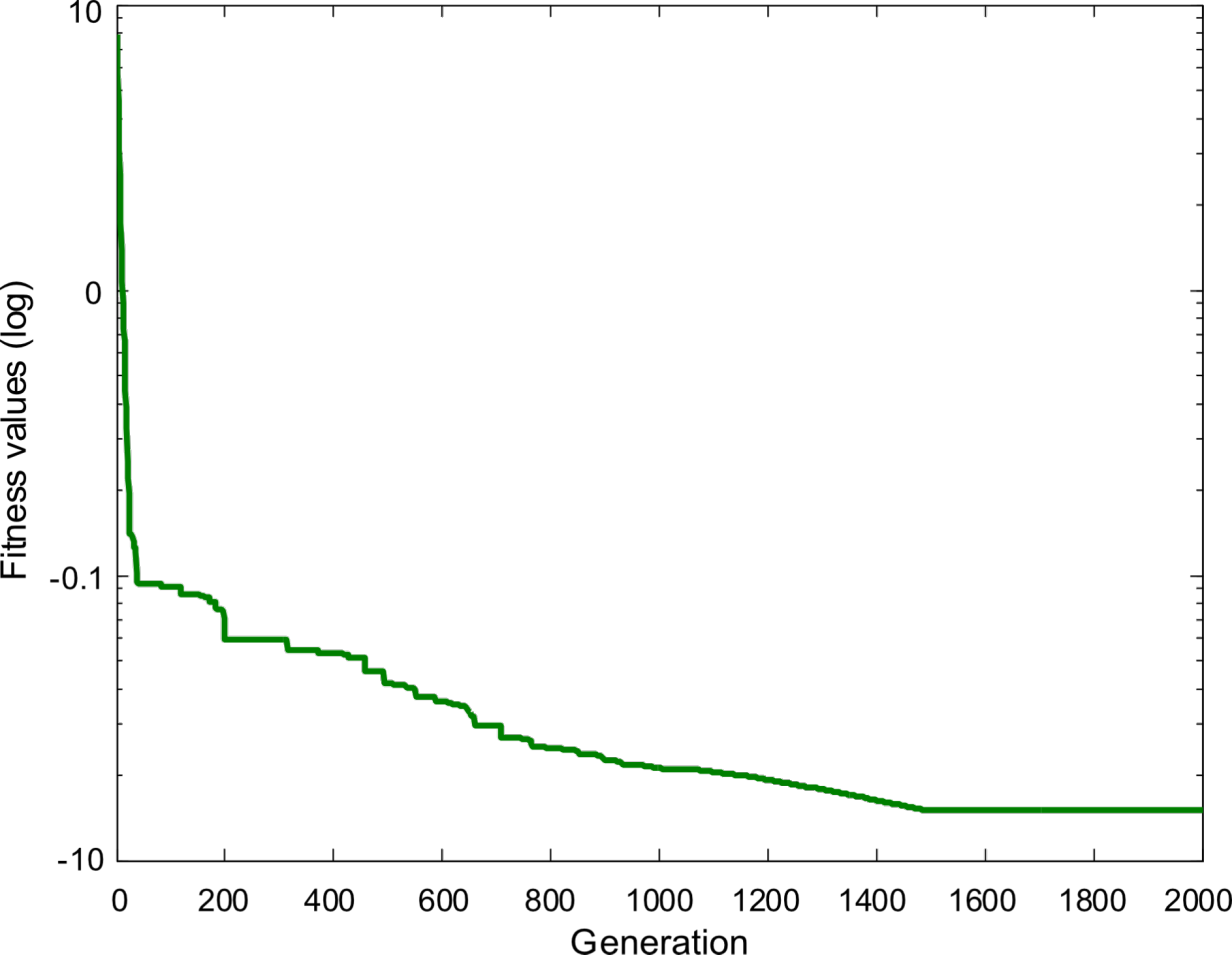
The convergence profile of the optimization of the proposed model using ACO technique.

The logarithmic convergence profile of the best fitness value obtained is plotted in Figure 6. The graph indicates that the algorithm converges to the optimized values with an acceptable convergence speed after around 1500 iterations.

It is apparent that there is a favorable agreement between the optimized proposed model of graphene-based EGFETs device and experimental result. It can be concluded that, the presented model can be applied as a powerful tool to optimize the graphene-based EGFETs device performance.

## Conclusion

Graphene as a 2D sheet of hexagonally arranged carbon atoms exhibits amazing carrier transport properties and a high sensitivity at the single-molecule level, which makes it a promising material for nanoscale devices. According to the graphene structure, it can satisfy the major requirements of a channel in electrolyte-gated transistor (EGFET) devices due to its ballistic transport, high conductivity, and strong mechanical and elasticity properties. An analytical modeling of the graphene capacitance as a major characteristic of EGFET is studied in this paper and the electrical circuit of the device is discussed. An EGFET based structure is employed as a platform and the graphene capacitance is studied. In order to enhance the accuracy of the proposed model, an ant colony optimization (ACO) algorithm is implemented and we obtained acceptable results with more than 97% of accuracy. Finally, for the purpose of verification, the *C*–*V* characteristic of the optimized model is investigated with an existing experimental study and shows an acceptable agreement. This paper demonstrates how the optimized model can be used to predict the capacitance variation of graphene in graphene-based devices.
